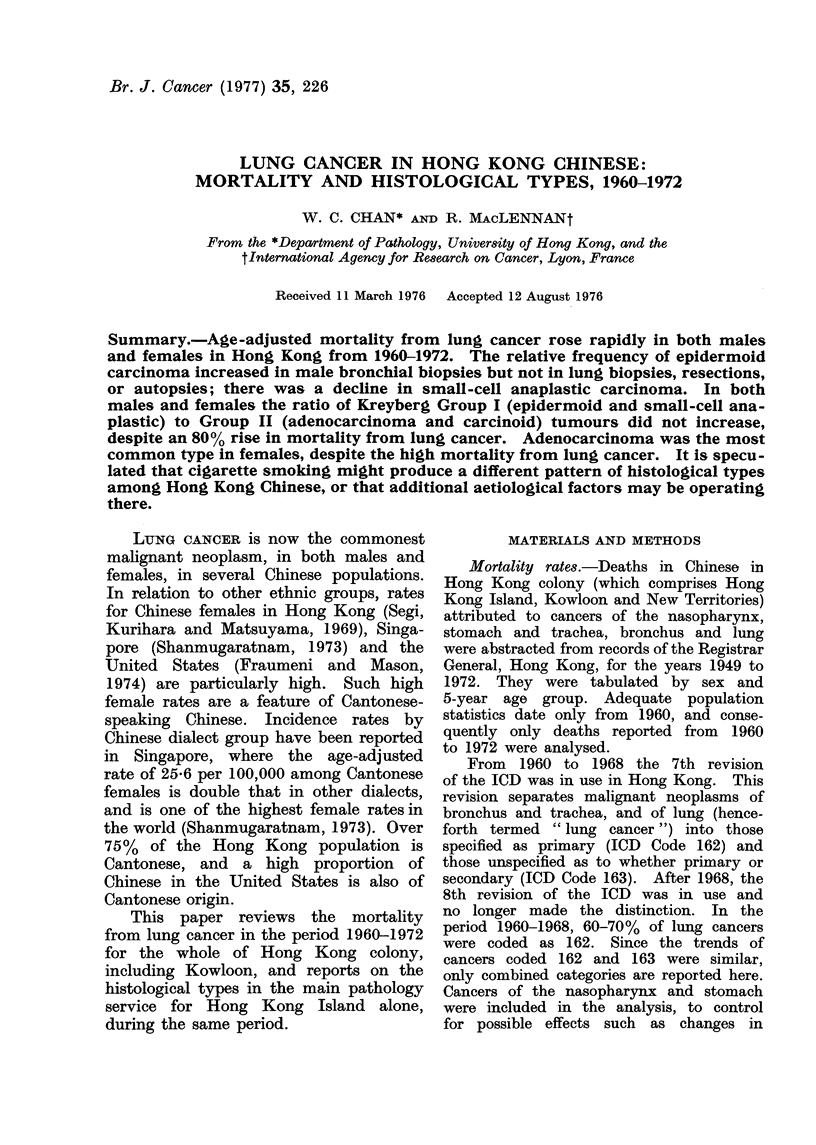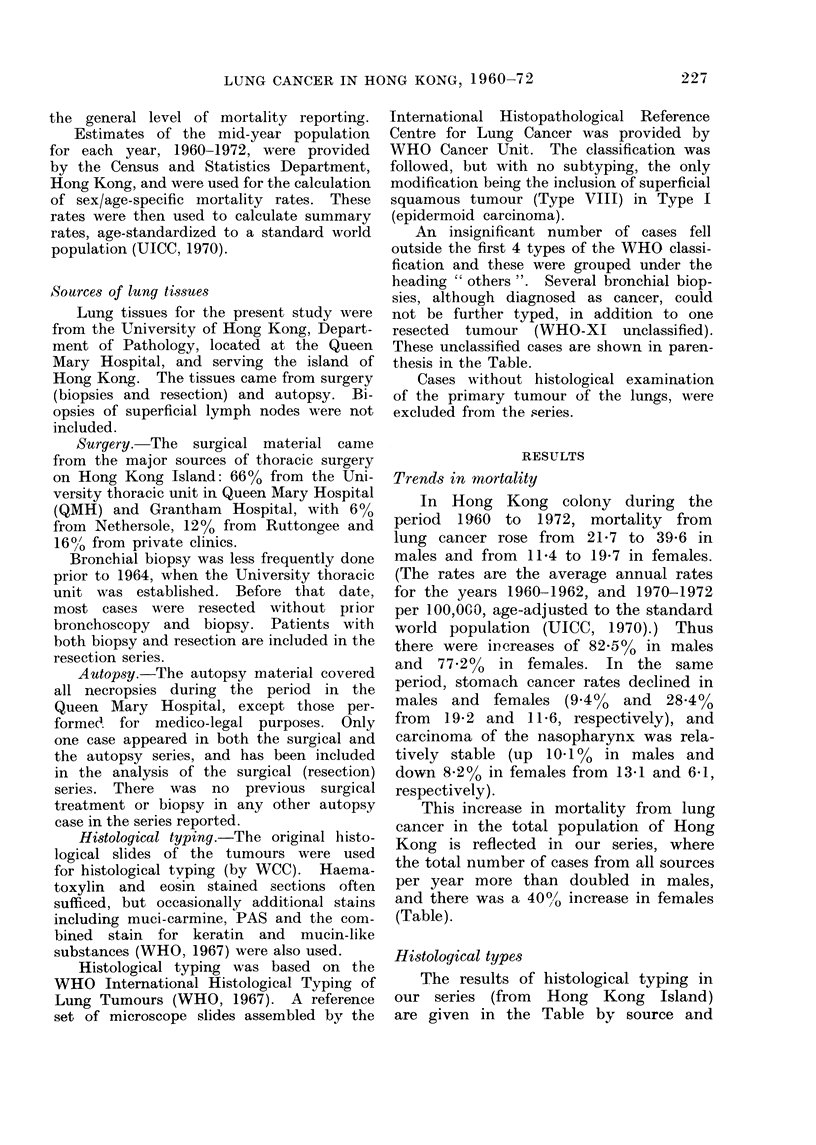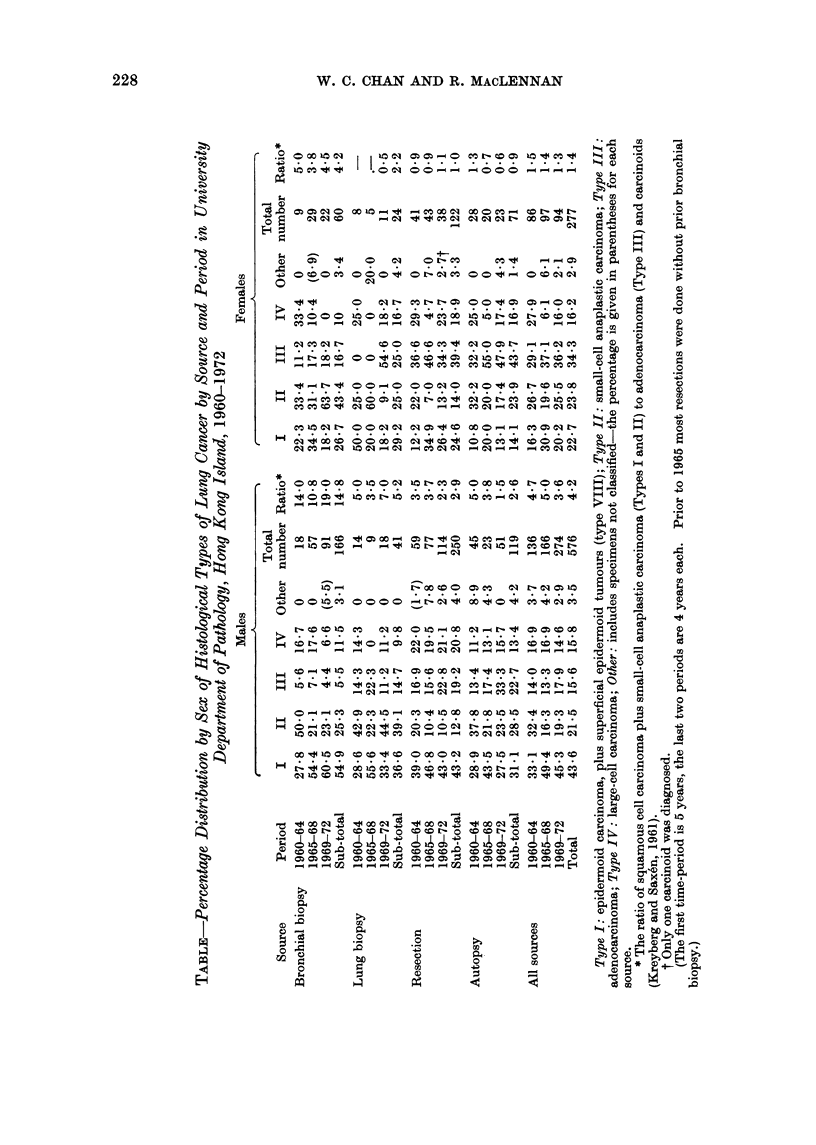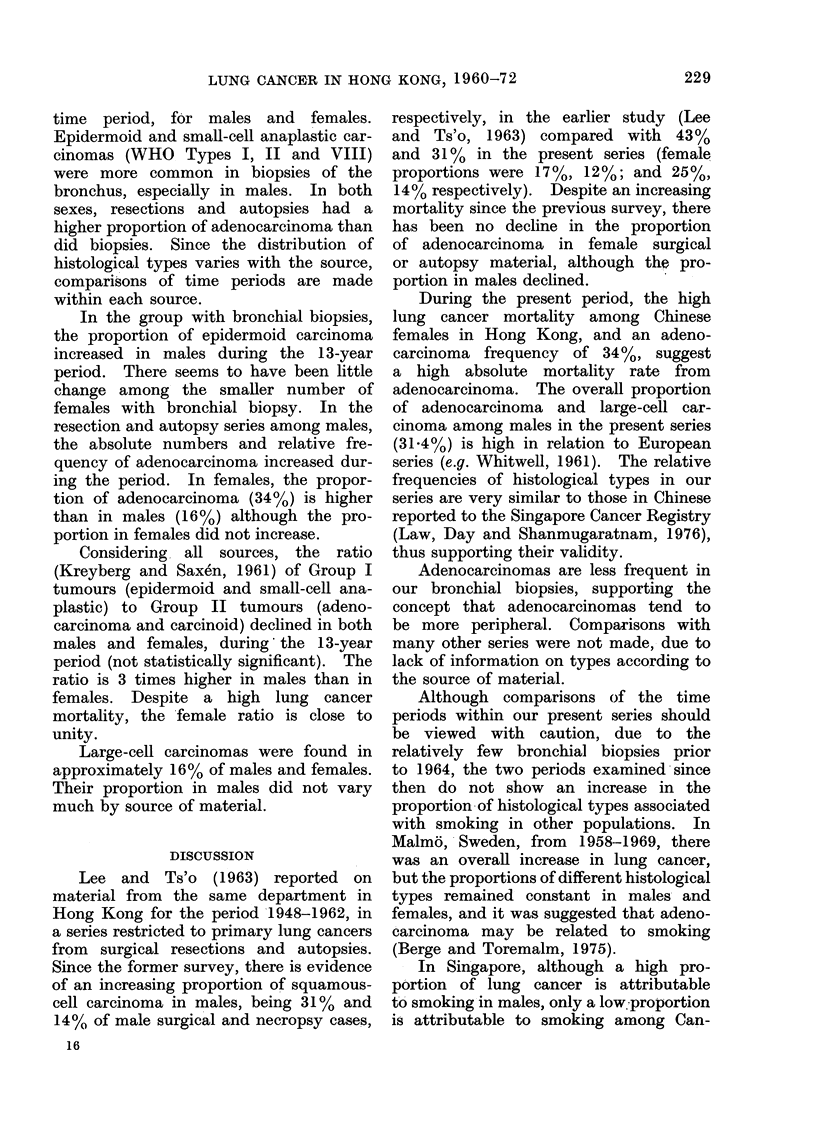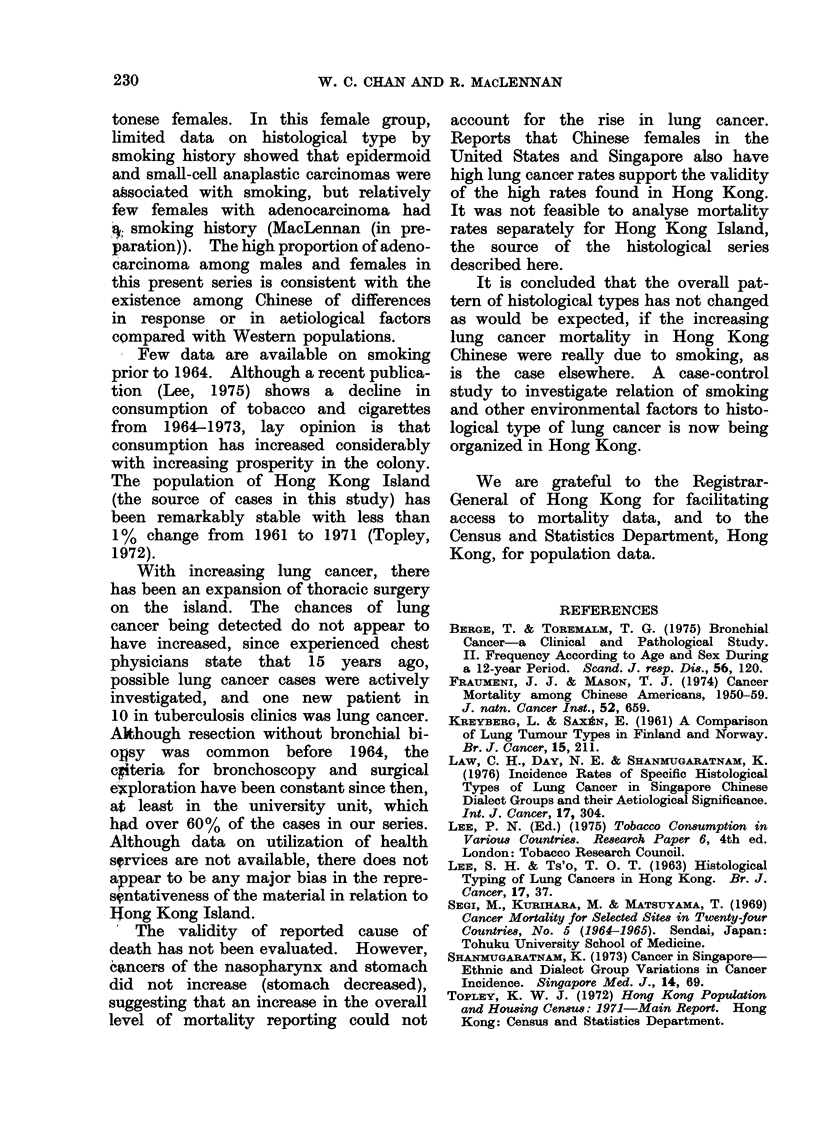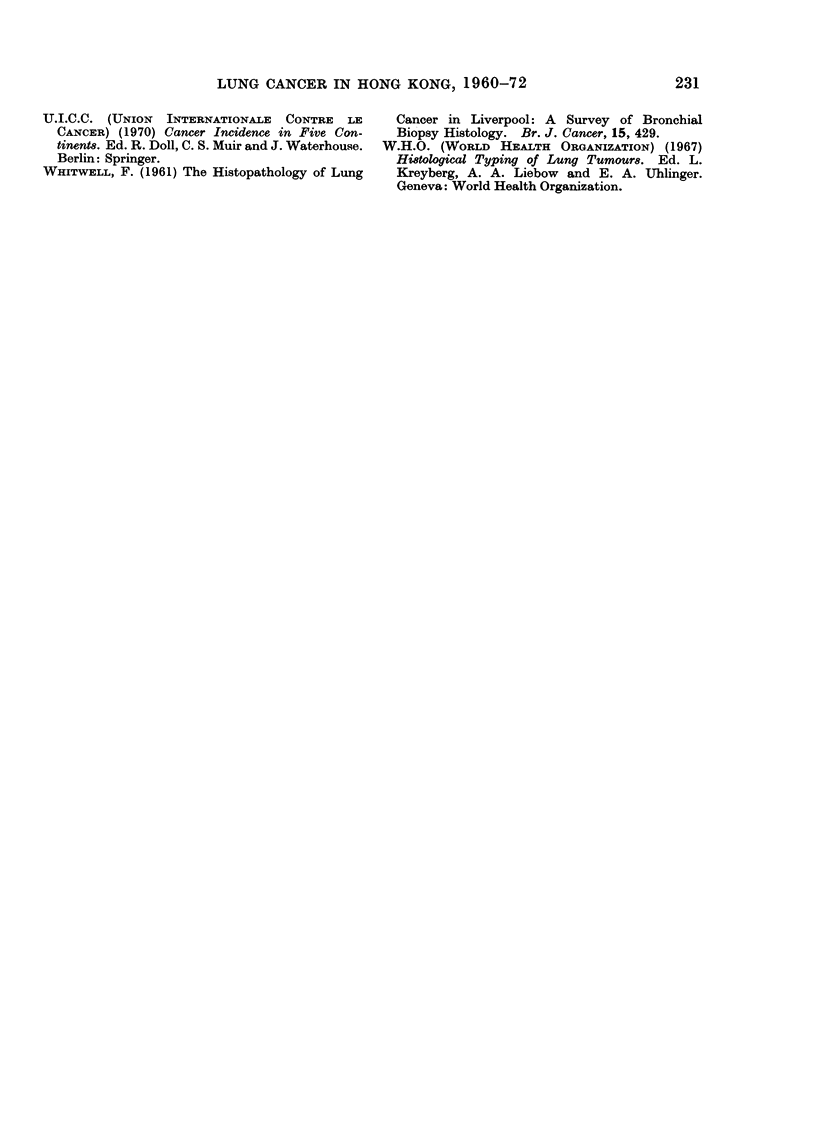# Lung cancer in Hong Kong Chinese: mortality and histological types, 1960-1972.

**DOI:** 10.1038/bjc.1977.30

**Published:** 1977-02

**Authors:** W. C. Chan, R. MacLennan

## Abstract

Age-adjusted mortality from lung cancer rose rapidly in both males and females in Hong Kong from 1960-1972. The relative frequency of epidermoid carcinoma increased in male bronchial biopsies but not in lung biopsies, resections, or autopsies; there was a decline in small-cell anaplastic carcinoma. In both males and females the ratio of Kreyberg Group I (epidermoid and small-cell anaplastic) to Group II (adenocarcinoma and carcinoid) tumours did not increase, despite an 80% rise in mortality from lung cancer. Adenocarcinoma was the most common type in females, despite the high mortality from lung cancer. It is speculated that cigarette smoking might produce a different pattern of histological types among Hong Kong Chinese, or that additional aetiological factors may be operating there.


*Br. J. Cancer* (1977) **35**, 226

### LUNG CANCER IN HONG KONG CHINESE: MORTALITY AND HISTOLOGICAL TYPES, 1960–1972

W. C. CHAN* AND R. MACLENNAN†

*From the *Department of Pathology, University of Hong Kong, and the †International Agency for Research on Cancer, Lyon, France*

Received 11 March 1976  Accepted 12 August 1976

**Summary.—Age-adjusted mortality from lung cancer rose rapidly in both males and females in Hong Kong from 1960–1972. The relative frequency of epidermoid carcinoma increased in male bronchial biopsies but not in lung biopsies, resections, or autopsies; there was a decline in small-cell anaplastic carcinoma. In both males and females the ratio of Kreyberg Group I (epidermoid and small-cell anaplastic) to Group II (adenocarcinoma and carcinoid) tumours did not increase, despite an 80% rise in mortality from lung cancer. Adenocarcinoma was the most common type in females, despite the high mortality from lung cancer. It is speculated that cigarette smoking might produce a different pattern of histological types among Hong Kong Chinese, or that additional aetiological factors may be operating there.**

LUNG CANCER is now the commonest malignant neoplasm, in both males and females, in several Chinese populations. In relation to other ethnic groups, rates for Chinese females in Hong Kong (Segi, Kurihara and Matsuyama, 1969), Singapore (Shanmugaratnam, 1973) and the United States (Fraumeni and Mason, 1974) are particularly high. Such high female rates are a feature of Cantonese-speaking Chinese. Incidence rates by Chinese dialect group have been reported in Singapore, where the age-adjusted rate of 25·6 per 100,000 among Cantonese females is double that in other dialects, and is one of the highest female rates in the world (Shanmugaratnam, 1973). Over 75% of the Hong Kong population is Cantonese, and a high proportion of Chinese in the United States is also of Cantonese origin.

This paper reviews the mortality from lung cancer in the period 1960–1972 for the whole of Hong Kong colony, including Kowloon, and reports on the histological types in the main pathology service for Hong Kong Island alone, during the same period.

### MATERIALS AND METHODS

*Mortality rates.*—Deaths in Chinese in Hong Kong colony (which comprises Hong Kong Island, Kowloon and New Territories) attributed to cancers of the nasopharynx, stomach and trachea, bronchus and lung were abstracted from records of the Registrar General, Hong Kong, for the years 1949 to 1972. They were tabulated by sex and 5-year age group. Adequate population statistics date only from 1960, and consequently only deaths reported from 1960 to 1972 were analysed.

From 1960 to 1968 the 7th revision of the ICD was in use in Hong Kong. This revision separates malignant neoplasms of bronchus and trachea, and of lung (henceforth termed "lung cancer") into those specified as primary (ICD Code 162) and those unspecified as to whether primary or secondary (ICD Code 163). After 1968, the 8th revision of the ICD was in use and no longer made the distinction. In the period 1960–1968, 60–70% of lung cancers were coded as 162. Since the trends of cancers coded 162 and 163 were similar, only combined categories are reported here. Cancers of the nasopharynx and stomach were included in the analysis, to control for possible effects such as changes in the general level of mortality reporting.

Estimates of the mid-year population for each year, 1960–1972, were provided by the Census and Statistics Department, Hong Kong, and were used for the calculation of sex/age-specific mortality rates. These rates were then used to calculate summary rates, age-standardized to a standard world population (UICC, 1970).

### *Sources of lung tissues*

Lung tissues for the present study were from the University of Hong Kong, Department of Pathology, located at the Queen Mary Hospital, and serving the island of Hong Kong. The tissues came from surgery (biopsies and resection) and autopsy. Biopsies of superficial lymph nodes were not included.

*Surgery.*—The surgical material came from the major sources of thoracic surgery on Hong Kong Island: 66% from the University thoracic unit in Queen Mary Hospital (QMH) and Grantham Hospital, with 6% from Nethersole, 12% from Ruttongee and 16% from private clinics.

Bronchial biopsy was less frequently done prior to 1964, when the University thoracic unit was established. Before that date, most cases were resected without prior bronchoscopy and biopsy. Patients with both biopsy and resection are included in the resection series.

*Autopsy.*—The autopsy material covered all necropsies during the period in the Queen Mary Hospital, except those performed for medico-legal purposes. Only one case appeared in both the surgical and the autopsy series, and has been included in the analysis of the surgical (resection) series. There was no previous surgical treatment or biopsy in any other autopsy case in the series reported.

*Histological typing.*—The original histological slides of the tumours were used for histological typing (by WCC). Haematoxylin and eosin stained sections often sufficed, but occasionally additional stains including muci-carmine, PAS and the combined stain for keratin and mucin-like substances (WHO, 1967) were also used.

Histological typing was based on the WHO International Histological Typing of Lung Tumours (WHO, 1967). A reference set of microscope slides assembled by the International Histopathological Reference Centre for Lung Cancer was provided by WHO Cancer Unit. The classification was followed, but with no subtyping, the only modification being the inclusion of superficial squamous tumour (Type VIII) in Type I (epidermoid carcinoma).

An insignificant number of cases fell outside the first 4 types of the WHO classification and these were grouped under the heading " others ". Several bronchial biopsies, although diagnosed as cancer, could not be further typed, in addition to one resected tumour (WHO-XI unclassified). These unclassified cases are shown in parenthesis in the Table.

Cases without histological examination of the primary tumour of the lungs, were excluded from the series.

### RESULTS

### *Trends in mortality*

In Hong Kong colony during the period 1960 to 1972, mortality from lung cancer rose from 21·7 to 39·6 in males and from 11·4 to 19·7 in females. (The rates are the average annual rates for the years 1960–1962, and 1970–1972 per 100,000, age-adjusted to the standard world population (UICC, 1970).) Thus there were increases of 82·5% in males and 77·2% in females. In the same period, stomach cancer rates declined in males and females (9·4% and 28·4% from 19·2 and 11·6, respectively), and carcinoma of the nasopharynx was relatively stable (up 10·1% in males and down 8·2% in females from 13·1 and 6·1, respectively).

This increase in mortality from lung cancer in the total population of Hong Kong is reflected in our series, where the total number of cases from all sources per year more than doubled in males, and there was a 40% increase in females (Table).

### *Histological types*

The results of histological typing in our series (from Hong Kong Island) are given in the Table by source and time period, for males and females. Epidermoid and small-cell anaplastic carcinomas (WHO Types I, II and VIII) were more common in biopsies of the bronchus, especially in males. In both sexes, resections and autopsies had a higher proportion of adenocarcinoma than did biopsies. Since the distribution of histological types varies with the source, comparisons of time periods are made within each source.

In the group with bronchial biopsies, the proportion of epidermoid carcinoma increased in males during the 13-year period. There seems to have been little change among the smaller number of females with bronchial biopsy. In the resection and autopsy series among males, the absolute numbers and relative frequency of adenocarcinoma increased during the period. In females, the proportion of adenocarcinoma (34%) is higher than in males (16%) although the proportion in females did not increase.

Considering all sources, the ratio (Kreyberg and Saxén, 1961) of Group I tumours (epidermoid and small-cell anaplastic) to Group II tumours (adenocarcinoma and carcinoid) declined in both males and females, during the 13-year period (not statistically significant). The ratio is 3 times higher in males than in females. Despite a high lung cancer mortality, the female ratio is close to unity.

Large-cell carcinomas were found in approximately 16% of males and females. Their proportion in males did not vary much by source of material.

### DISCUSSION

Lee and Ts'o (1963) reported on material from the same department in Hong Kong for the period 1948–1962, in a series restricted to primary lung cancers from surgical resections and autopsies. Since the former survey, there is evidence of an increasing proportion of squamous-cell carcinoma in males, being 31% and 14% of male surgical and necropsy cases, respectively, in the earlier study (Lee and Ts'o, 1963) compared with 43% and 31% in the present series (female proportions were 17%, 12%; and 25%, 14% respectively). Despite an increasing mortality since the previous survey, there has been no decline in the proportion of adenocarcinoma in female surgical or autopsy material, although the proportion in males declined.

During the present period, the high lung cancer mortality among Chinese females in Hong Kong, and an adenocarcinoma frequency of 34%, suggest a high absolute mortality rate from adenocarcinoma. The overall proportion of adenocarcinoma and large-cell carcinoma among males in the present series (31·4%) is high in relation to European series (*e.g.* Whitwell, 1961). The relative frequencies of histological types in our series are very similar to those in Chinese reported to the Singapore Cancer Registry (Law, Day and Shanmugaratnam, 1976), thus supporting their validity.

Adenocarcinomas are less frequent in our bronchial biopsies, supporting the concept that adenocarcinomas tend to be more peripheral. Comparisons with many other series were not made, due to lack of information on types according to the source of material.

Although comparisons of the time periods within our present series should be viewed with caution, due to the relatively few bronchial biopsies prior to 1964, the two periods examined since then do not show an increase in the proportion of histological types associated with smoking in other populations. In Malmö, Sweden, from 1958–1969, there was an overall increase in lung cancer, but the proportions of different histological types remained constant in males and females, and it was suggested that adenocarcinoma may be related to smoking (Berge and Toremalm, 1975).

In Singapore, although a high proportion of lung cancer is attributable to smoking in males, only a low proportion is attributable to smoking among Cantonese females. In this female group, limited data on histological type by smoking history showed that epidermoid and small-cell anaplastic carcinomas were associated with smoking, but relatively few females with adenocarcinoma had a smoking history (MacLennan (in preparation)). The high proportion of adenocarcinoma among males and females in this present series is consistent with the existence among Chinese of differences in response or in aetiological factors compared with Western populations.

Few data are available on smoking prior to 1964. Although a recent publication (Lee, 1975) shows a decline in consumption of tobacco and cigarettes from 1964–1973, lay opinion is that consumption has increased considerably with increasing prosperity in the colony. The population of Hong Kong Island (the source of cases in this study) has been remarkably stable with less than 1% change from 1961 to 1971 (Topley, 1972).

With increasing lung cancer, there has been an expansion of thoracic surgery on the island. The chances of lung cancer being detected do not appear to have increased, since experienced chest physicians state that 15 years ago, possible lung cancer cases were actively investigated, and one new patient in 10 in tuberculosis clinics was lung cancer. Although resection without bronchial biopsy was common before 1964, the criteria for bronchoscopy and surgical exploration have been constant since then, at least in the university unit, which had over 60% of the cases in our series. Although data on utilization of health services are not available, there does not appear to be any major bias in the representativeness of the material in relation to Hong Kong Island.

The validity of reported cause of death has not been evaluated. However, cancers of the nasopharynx and stomach did not increase (stomach decreased), suggesting that an increase in the overall level of mortality reporting could not account for the rise in lung cancer. Reports that Chinese females in the United States and Singapore also have high lung cancer rates support the validity of the high rates found in Hong Kong. It was not feasible to analyse mortality rates separately for Hong Kong Island, the source of the histological series described here.

It is concluded that the overall pattern of histological types has not changed as would be expected, if the increasing lung cancer mortality in Hong Kong Chinese were really due to smoking, as is the case elsewhere. A case-control study to investigate relation of smoking and other environmental factors to histological type of lung cancer is now being organized in Hong Kong.

We are grateful to the Registrar-General of Hong Kong for facilitating access to mortality data, and to the Census and Statistics Department, Hong Kong, for population data.

### REFERENCES

Berge, T. & Toremalm, T. G. (1975) Bronchial Cancer—a Clinical and Pathological Study. II. Frequency According to Age and Sex During a 12-year Period. *Scand. J. resp. Dis.*, **56**, 120.

Fraumeni, J. J. & Mason, T. J. (1974) Cancer Mortality among Chinese Americans, 1950–59. *J. natn. Cancer Inst.*, **52**, 659.

Kreyberg, L. & Saxén, E. (1961) A Comparison of Lung Tumour Types in Finland and Norway. *Br. J. Cancer*, **15**, 211.

Law, C. H., Day, N. E. & Shanmugaratnam, K. (1976) Incidence Rates of Specific Histological Types of Lung Cancer in Singapore Chinese Dialect Groups and their Aetiological Significance. *Int. J. Cancer*, **17**, 304.

Lee, P. N. (Ed.) (1975) *Tobacco Consumption in Various Countries. Research Paper 6*, 4th ed. London: Tobacco Research Council.

Lee, S. H. & Ts'o, T. O. T. (1963) Histological Typing of Lung Cancers in Hong Kong. *Br. J. Cancer*, **17**, 37.

Segi, M., Kurihara, M. & Matsuyama, T. (1969) *Cancer Mortality for Selected Sites in Twenty-four Countries, No. 5 (1964–1965)*. Sendai, Japan: Tohuku University School of Medicine.

Shanmugaratnam, K. (1973) Cancer in Singapore—Ethnic and Dialect Group Variations in Cancer Incidence. *Singapore Med. J.*, **14**, 69.

Topley, K. W. J. (1972) *Hong Kong Population and Housing Census: 1971—Main Report*. Hong Kong: Census and Statistics Department.

U.I.C.C. (UNION INTERNATIONALE CONTRE LE CANCER) (1970) *Cancer Incidence in Five Continents*. Ed. R. Doll, C. S. Muir and J. Waterhouse. Berlin: Springer.

WHITWELL, F. (1961) The Histopathology of Lung Cancer in Liverpool: A Survey of Bronchial Biopsy Histology. *Br. J. Cancer*, **15**, 429.

W.H.O. (WORLD HEALTH ORGANIZATION) (1967) *Histological Typing of Lung Tumours*. Ed. L. Kreyberg, A. A. Liebow and E. A. Uhlinger. Geneva: World Health Organization.